# Sickle cell anaemia and severe *Plasmodium falciparum* malaria: a secondary analysis of the Transfusion and Treatment of African Children Trial (TRACT)

**DOI:** 10.1016/S2352-4642(22)00153-5

**Published:** 2022-07-02

**Authors:** Sophie Uyoga, Peter Olupot-Olupot, Roisin Connon, Sarah Kiguli, Robert O Opoka, Florence Alaroker, Rita Muhindo, Alexander W Macharia, Arjen M Dondorp, Diana M Gibb, A Sarah Walker, Elizabeth C George, Kathryn Maitland, Thomas N Williams

**Affiliations:** KEMRI-Wellcome Trust Research Programme, Kilifi, Kenya; Busitema University Faculty of Health Sciences, Mbale Regional Referral Hospital, Mbale, Uganda; Mbale Clinical Research Institute, Mbale, Uganda; Medical Research Council Clinical Trials Unit (MRC CTU) at University College London, London, UK; Department of Paediatrics and Child Health, School of Medicine, Makerere University, Kampala, Uganda; Department of Paediatrics and Child Health, School of Medicine, Makerere University, Kampala, Uganda; Soroti Regional Referral Hospital, Soroti, Uganda; Mbale Clinical Research Institute, Mbale, Uganda; KEMRI-Wellcome Trust Research Programme, Kilifi, Kenya; Mahidol-Oxford Research Unit, Faculty of Tropical Medicine, Mahidol University, Bangkok, Thailand; Medical Research Council Clinical Trials Unit (MRC CTU) at University College London, London, UK; Medical Research Council Clinical Trials Unit (MRC CTU) at University College London, London, UK; Medical Research Council Clinical Trials Unit (MRC CTU) at University College London, London, UK; KEMRI-Wellcome Trust Research Programme, Kilifi, Kenya; Department of Infectious Disease and Institute of Global Health Innovation, Division of Medicine, Imperial College London, London, UK; KEMRI-Wellcome Trust Research Programme, Kilifi, Kenya; Department of Infectious Disease and Institute of Global Health Innovation, Division of Medicine, Imperial College London, London, UK

## Abstract

**Background:**

Sickle cell anaemia (SCA) has historically been associated with high levels of childhood mortality in Africa. Although malaria has a major contribution to this mortality, to date, the clinical pathology of malaria among children with SCA has been poorly described. We aimed to explore the relationship between SCA and *Plasmodium falciparum* malaria in further detail by investigating the burden and severity of malaria infections among children recruited with severe anaemia to the TRACT trial of blood transfusion in Africa.

**Methods:**

This study is a post-hoc secondary analysis of the TRACT trial data, conducted after trial completion. TRACT was an open-label, multicentre, factorial, randomised controlled trial enrolling children aged 2 months to 12 years who presented with severe anaemia (haemoglobin <6·0 g/dL) to four hospitals in Africa. This secondary analysis is restricted to Uganda, where the birth prevalence of SCA is approximately 1% and malaria transmission is high. Children were classified as normal (HbAA), heterozygous (HbAS), or homozygous (HbSS; SCA) for the rs334 A→T sickle mutation in *HBB* following batch-genotyping by PCR at the end of the trial. To avoid confounding from SCA-specific medical interventions, we considered children with an existing diagnosis of SCA (known SCA) separately from those diagnosed at the end of the trial (unknown SCA). The outcomes considered in this secondary analysis were measures of *P falciparum* parasite burden, features of severe malaria, and mortality at day 28 in malaria-positive children.

**Findings:**

Between Sept 17, 2014, and May 15, 2017, 3944 children with severe anaemia were enrolled into the TRACT trial. 3483 children from Uganda were considered in this secondary analysis. Overall, 1038 (30%) of 3483 Ugandan children had SCA. 1815 (78%) of 2321 children without SCA (HbAA) tested positive for *P falciparum* malaria, whereas the prevalence was significantly lower in children with SCA (347 [33%] of 1038; p<0·0001). Concentrations of plasma *P falciparum* histidine-rich protein 2 (PfHRP2), a marker of the total burden of malaria parasites within an individual, were significantly lower in children with either known SCA (median 8 ng/mL; IQR 0–57) or unknown SCA (7 ng/mL; 0–50) than in HbAA children (346 ng/mL; 21–2121; p<0·0001). In contrast to HbAA children, few HbSS children presented with classic features of severe and complicated malaria, but both the frequency and severity of anaemia were higher in HbSS children. We found no evidence for increased mortality at day 28 in those with SCA compared with those without SCA overall (hazard ratios 1·07 [95% CI 0·31–3·76] for known SCA and 0·67 [0·15–2·90] for unknown SCA).

**Interpretation:**

The current study suggests that children with SCA are innately protected against classic severe malaria. However, it also shows that even low-level infections can precipitate severe anaemic crises that would likely prove fatal without rapid access to blood transfusion services.

**Funding:**

UK Medical Research Council, Wellcome, and UK National Institute for Health and Care Research.

## Introduction

Sickle cell anaemia (SCA) is the commonest life-threatening genetic disorder worldwide. The causal mutation, the rs334 A→T single-nucleotide polymorphism in *HBB*, is the classic example of a balanced polymorphism, where current allele frequencies reflect a survival advantage in carriers and increased mortality in homozygotes.^1^ There is now abundant evidence to support a central role for *Plasmodium falciparum* malaria in the positive selection of heterozygotes (HbAS)^2–5^ but its place in the negative selection of SCA (HbSS) is less clear.^6^

It is often stated that severe malaria is a major reason for the high mortality seen in children with SCA in Africa.^7–9^ Severe and complicated malaria is defined on the basis of a range of specific clinical and laboratory features in the presence of *P falciparum* parasitaemia.^10^ The commonest forms of severe malaria among children in Africa are cerebral malaria, respiratory distress (caused by deep or acidotic breathing), and severe malaria anaemia.^10^ Similar clinical features can develop in children with SCA who do not have malaria,^11^ which can make it difficult to attribute such features to malaria. Measurement of the parasite-derived enzyme *P falciparum* histidine-rich protein 2 (PfHRP2),^12,13^ which reflects the burden of parasites that lie sequestered in the microcirculation,^14^ could potentially be helpful in this regard. Recent studies in children without SCA have shown that plasma PfHRP2 can help clinicians to distinguish true severe malaria from severe illnesses of an alternative cause in children who also happen to be carrying malaria parasites incidentally.^15–17^

In the current study, we conducted a secondary analysis of data from the Transfusion and Treatment of severe anaemia in African Children Trial (TRACT^18^), in which we also included measurements of PfHRP2 concentrations, to explore the relationship between SCA and malaria in further detail.

## Methods

### Study design and participants

The current study is a post-hoc secondary analysis of data from the TRACT trial, conducted after the trial was completed. The TRACT trial was an open-label, multicentre, factorial, randomised controlled trial enrolling children aged 2 months to 12 years who presented with severe anaemia (haemoglobin <6·0 g/dL) to four hospitals in Africa. Full details of the trial have been published previously.^19–21^ TRACT was registered (ISRCTN84086586) on Feb 11, 2013. The trial flow is detailed in the appendix (p 4). Briefly, TRACT investigated the management of both complicated and uncomplicated severe anaemia (haemoglobin <6·0 g/dL) among children in Uganda and Malawi. Data were collected on structured case report forms that were completed at admission and at reviews every 30 min during transfusion, then regularly (at 2 h, 4 h, 8 h, 16 h, 24 h, and daily thereafter) during admission. After discharge, children were routinely followed up on days 28, 90, and 180 after randomisation for review of clinical status and haemoglobin measurements. Serious adverse events were actively solicited at every assessment. Quality control of data was done through monitoring visits and data checks performed centrally by the trial statistician (ASW and ECG). We restricted our current analysis to Uganda because the low birth prevalence of SCA in Malawi meant that only six (1·3%) of 461 children recruited there had either known or unknown SCA, as anticipated from the low national allele frequency data for the country.^22^ In Uganda, the trial was conducted in three hospitals, two in the malaria hyperendemic Eastern region (Mbale and Soroti^23^), and Mulago Hospital in Kampala, where transmission is mesoendemic.^24^ The background prevalence of SCA during infancy in these regions has recently been estimated at 1·2% and 0·7%, respectively.^25^

Eligibility for the complicated anaemia stratum required the presence of one or more of the following features of clinical severity: profound anaemia (haemoglobin <4·0 g/dL), impaired consciousness, increased work of breathing, blackwater fever, or known SCA. Children recruited to this stratum were randomly assigned to receive either 20 mL/kg or 30 mL/kg of whole blood, or the equivalent volumes (10 mL/kg or 15 mL/kg) of packed red blood cells. Children were eligible for recruitment to the uncomplicated anaemia stratum if their haemoglobin concentration was between 4·0 g/dL and 6·0 g/dL and they had none of the aforementioned severity features. In this stratum, children were randomly assigned to receive either no immediate transfusion or immediate transfusion of 20 mL/kg or 30 mL/kg of whole blood or the equivalent volumes (10 mL/kg or 15 mL/kg) of packed cells. Children with known SCA were ineligible for recruitment to this stratum because the investigators lacked equipoise regarding the ethics of withholding transfusions from children with known SCA and a haemoglobin concentration of less than 6·0 g/dL. All children were followed up until day 28 for the primary endpoint, mortality, and to day 180 for a range of additional events that included readmission and death.

Written informed consent for the trial was sought from all parents or guardians, but where not logistically possible, ethics committees approved verbal assent with delayed consent as soon as practicable.^26^ This process included consent to the storage and reuse of samples and data for secondary analyses such as this. The protocol was approved by ethics committees at Imperial College London, UK (ICREC_13-1-11) and Makerere University, Uganda (SOMREC 2013-050).

### Procedures

Full details of the management and conduct of the trial have been published previously.^20^ Briefly, a structured clinical case report form and baseline investigations were completed for all participants at admission, and therefore before transfusion. Full blood counts and biochemical measurements were conducted in accredited laboratories following standard operating procedures. Malaria was diagnosed using the Paracheck rapid diagnostic test (RDT; Orchid Biomedical Systems, Goa, India), which is based on the detection of PfHRP2 in whole blood, and through the duplicate examination of Giemsa-stained peripheral blood films by trained microscopists. All samples were stored and transported at 4°C until processing and all samples for archiving were frozen at –80°C within 24 h of collection. On conclusion of the trial, all participants were batch-genotyped for the rs334 A→T *HBB* mutation by PCR,^27^ and plasma concentrations of PfHRP2 were measured by ELISA as previously described.^15^ The latter is distinct from the whole-blood PfHRP2 rapid test, which is highly sensitive but non-quantitative. Both SCA genotyping and plasma PfHRP2 quantification were conducted on previously frozen and archived samples collected at the time of participant recruitment, before the receipt of any transfusions.^20^ We did not test for other major forms of sickle cell disease caused by the coinheritance of HbS with mutations causing HbC or β^0^-thalassaemia because neither have been found in previous studies conducted in the same population.^25,28^

### Outcomes

The outcomes considered in this secondary analysis were measures of *P falciparum* parasite burden, features of severe malaria, and mortality at day 28 in malaria-positive children. Measures of *P falciparum* parasite burden were parasite density and plasma PfHRP2 concentration. 11 features of severe malaria were considered, including profound anaemia (haemoglobin <4·0 g/dL), hyper- parasitaemia (>100 000 *P falciparum* parasites per μL), and plasma PfHRP2 concentration of more than 1000 ng/mL.

### Statistical analysis

We classified children as either: (1) normal (HbAA), (2) heterozygous (sickle cell trait; HbAS), or (3) homozygous (HbSS; SCA) for the rs334 A→T mutation. The homozygous group were further classified into those with an existing diagnosis (known SCA) and those whose diagnosis was only revealed by genotyping at the end of the trial (unknown SCA). Because patients with known SCA were excluded from one arm of the trial, and because they are often managed differently in terms of their clinical care (such as the routine receipt of antimalarial chemoprophylaxis and other malaria-preventive measures), we analysed data on known and unknown SCA groups separately to avoid potential problems with interpretation. We compared baseline characteristics in malaria-positive trial participants by SCA group using Mann-Whitney U tests or Fisher’s exact tests for continuous and categorical factors, respectively, and used histograms to examine the distributions. PfHRP2 was also categorised as less than 1000 ng/mL or more than (or equal to) 1000 ng/mL, a value that has been used to distinguish children with true severe malaria from those with severe illnesses in the presence of incidental parasitaemia in other studies.^15,29^ We investigated the association of SCA group and mortality at day 28 using a previously developed Cox proportional hazards model for predictors of mortality, which adjusted for the centre of recruitment, transfusion volume randomisation, temperature, and the interaction between transfusion volume and temperature. Other potential confounders were identified using backwards elimination with a threshold p value of 0·1, and included as covariates in the models. Candidate covariates, which were identified using clinical input, previous knowledge, and through review of the literature, were demographic details, vital signs on admission, clinical history of the illness, results of laboratory tests at admission, and information on the trial arm and trial transfusions. Continuous variables were tested for linearity by modelling as fractional polynomials with α=0·05. All analyses were conducted using Stata, version 16.1.

### Role of the funding source

The funder of the study had no role in study design, data collection, data analysis, data interpretation, or writing of the report.

## Results

**Figure 1:**
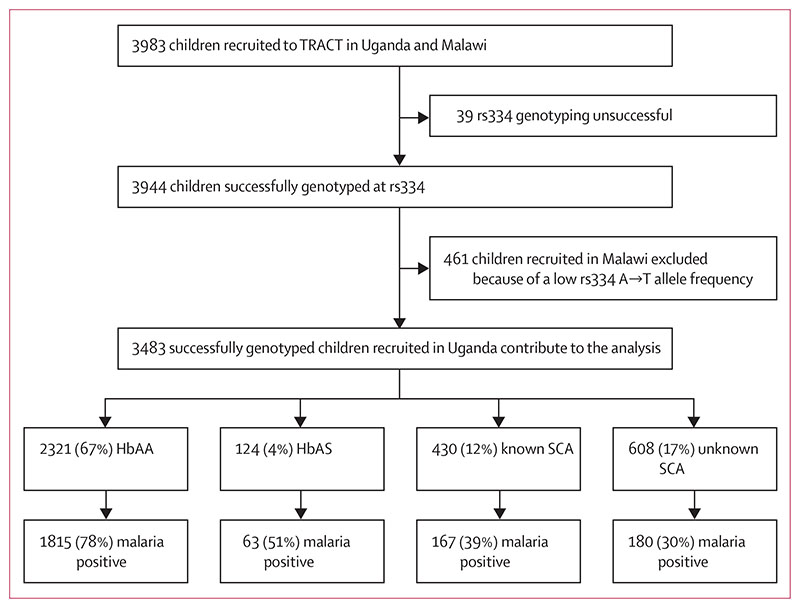
TRACT recruitment profile for this secondary analysis SCA=sickle cell anaemia.

The recruitment profile for the TRACT trial is summarised in figure 1. 3199 children with severe anaemia were enrolled into the TRACT trial between Sept 17, 2014, and May 15, 2017. Of the 3483 children enrolled and successfully genotyped in Uganda, 2321 (67%) had HbAA, 124 (4%) had HbAS, 430 (12%) had known SCA (disclosed at admission), and 608 (17%) had unknown SCA (totalling 1038 [30%] of 3483 with SCA overall).

2225 (64%) of the 3483 Ugandan participants tested positive for malaria at recruitment by either RDT or microscopy. Of these children, 1036 (47%) tested positive by both RDT and microscopy, 1136 (52%) were positive by RDT but negative by microscopy, suggestive of recovery from a recent malaria infection, and a small number (26 [1%]) fell into other categories (appendix p 1). When stratified by SCA genotype, 1815 (78%) of the 2321 HbAA children were malaria positive, whereas this proportion was significantly lower in those with HbAS (63 [51%] of 124; difference in proportion –27% [95% CI –36% to –19%]; χ^2^ p<0·0001) and lower still in those with either known SCA (167 [39%] of 430; difference *vs* HbAA –39% [–44% to –35%]; p<0·0001) or unknown SCA (180 [30%] of 608; difference *vs* HbAA –49% [–53% to –45%]; p<0·0001]; figure 1).

The clinical and laboratory characteristics of malaria-positive trial participants, stratified by SCA group, are summarised in table 1. Median age was similar across the groups except for the children with known SCA, who were approximately twice as old as those in the other three groups. Compared with HbAA children, median haemoglobin concentrations were marginally lower in those with either known SCA (4·3 g/dL [IQR 3·4–5·1] *vs* 4·5 [3·6–5·4] in HbAA; ranksum p=0·010) or unknown SCA (4·2 [3·5–4·9]; p=0·0004). In keeping with the haematology of SCA,^30^ median red cell volumes (measured as mean corpuscular volume) were significantly higher in both SCA groups. Plasma C-reactive protein concentrations were significantly lower, and platelet counts significantly higher, in children with either HbAS or SCA than in HbAA children (table 1).

**Figure 2:**
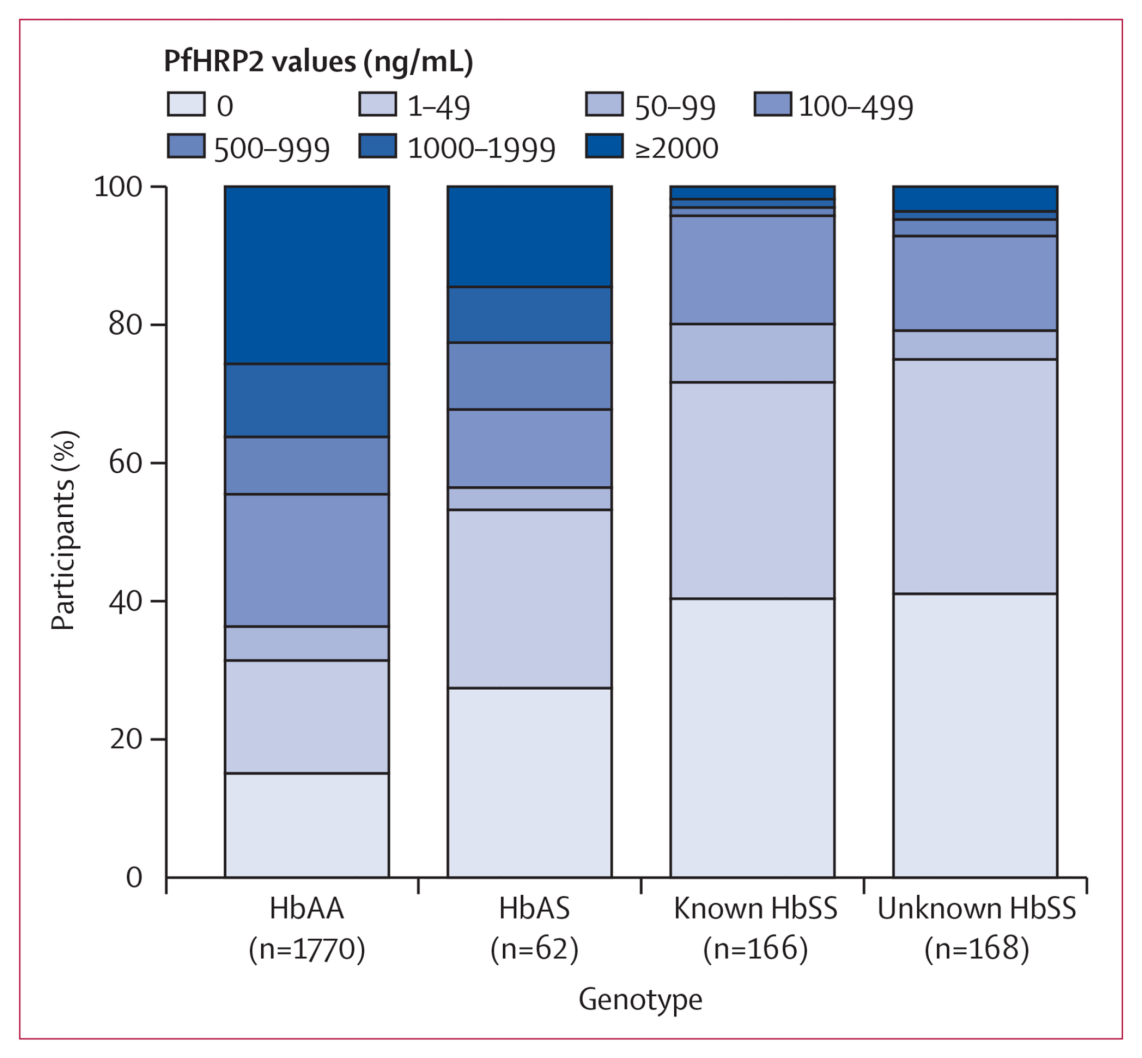
Distribution of PfHRP2 values by sickle genotype Data underlying the graph are shown in the appendix (p 3). PfHRP2=*Plasmodium falciparum* histidine-rich protein 2.

The features of malaria severity, stratified by SCA group, are summarised in table 2. Because children with features of clinical severity were only recruited into one stratum of the trial, we present these data stratified by study stratum. Median parasite densities were more than three times lower in both children with HbAS (data not shown) and those in both SCA groups than they were in those with HbAA overall (table 2). Similarly, median concentrations of plasma PfHRP2 were considerably lower in those with either known SCA (8 ng/mL; IQR 0–57) or unknown SCA (7 ng/mL; 0–50 ng/mL) than they were in HbAA children (346 ng/mL; IQR 21–2121; p<0·0001 for both comparisons). High plasma concentrations of PfHRP2 were only seen in a small proportion of children with SCA (figure 2 and appendix p 5). Concentrations of more than 1000 ng/mL were seen in 36% of HbAA children but in only 3–5% of children with SCA overall. Plasma PfHRP2 was undetectable in at least 40% of children in the combined SCA groups in comparison to only 15% of malaria-positive HbAA children. The proportions of malaria-positive children with no plasma PfHRP2 detectable by ELISA, stratified by the method of malaria diagnosis, are summarised in the appendix (p 1). Of note, plasma PfHRP2 was undetectable in only 85 (8%) of 1004 children testing positive by both RDT and microscopy, but in 326 (29%) of 1136 children who tested positive by RDT but negative by microscopy, and in nine (35%) of 26 children with other test–result combinations.

By design, most of the children presenting with clinical signs of severe malaria were recruited to the complicated stratum of the trial (table 2). Within this stratum, most of the children with unknown SCA (72 [75%] of 96) had profound anaemia (haemoglobin <4·0 g/dL). However, other severity features were significantly less frequent among the children with SCA overall except for renal impairment and hypoglycaemia, both of which were comparatively rare in those recruited overall.

The mortality to day 28 among the malaria-positive children, stratified by SCA category, is summarised in table 3. We found no evidence for increased mortality in those with SCA compared with those without SCA overall. However, on stratifying by plasma concentrations of PfHRP2 we found that all the deaths in the malaria-positive children with SCA occurred before day 7 and were limited to children with detectable concentrations of plasma PfHRP2 (appendix p 2). Mortality by day 7 occurred in two (25%) of eight children with unknown SCA with a plasma PfHRP2 value of more than 1000 ng/mL and in three (4%) of 81 children with known SCA who had plasma PfHRP2 concentrations of 10–100 ng/mL (appendix p 2).

## Discussion

Among 3483 children presenting with all-cause severe anaemia who were enrolled at the point of hospital admission into the TRACT trial in Uganda, 30% had SCA overall. In comparison to HbAA children, a significantly lower proportion of those with SCA tested positive for *P falciparum* malaria, and both total parasite burden and levels of inflammation, a correlate of clinical severity,^32^ were significantly lower in those with SCA who tested positive compared with HbAA children who tested positive. Only a small proportion of the children with SCA presented with the clinical or laboratory features of classic severe malaria.^10^ These observations suggest that malaria infections are both less frequent and less severe among children with SCA than they are in HbAA children. Only 4% of children recruited to TRACT had HbAS, in comparison to approximately 16% of children within the same community,^33^ reflecting the very high levels of malaria protection afforded by this condition.^2–5^

Given that steady state haemoglobin concentrations in children with SCA in Africa average only 6·0–8·0 g/dL,^30^ on first impression the high proportion of children with SCA might not seem particularly surprising. Importantly, however, more than three-quarters of all the malaria-positive children with SCA were recruited through the complicated stratum of the trial. Recruitment to this stratum required the presence of at least one severity feature, many of which would qualify as classic severe malaria^10^ in children without SCA. Many of the children recruited to this stratum of the trial were profoundly unwell, 10% being prostrated, 9% having respiratory distress, and 17% being profoundly acidotic; however, in marked contrast to those without SCA, very few of the children with unknown SCA had malaria infections that were characterised by either high parasite densities or by a high sequestered parasite load based on high PfHRP2 plasma concentrations. Notably, 75% of the malaria-positive children with unknown SCA were recruited with profound anaemia (haemoglobin <4·0 g/dL). Focusing specifically on the children with clinically defined cerebral malaria, median plasma PfHRP2 concentrations were more than 1000 ng/mL among the HbAA subgroup, levels consistent with those reported in previous studies.^15,34,35^ Conversely, concentrations were low or undetectable in all those in this same subgroup who had SCA. Classic cerebral malaria is related to the biomass of mature *P falciparum* parasites that are sequestered in the cerebral vasculature^36^ and, as a consequence, is associated with a high plasma PfHRP2 concentration.^15^ The very low concentrations found in the children with SCA within this subgroup means that in the majority, their impaired consciousness was probably not causally related to malaria. This notion accords with observations made in several previous studies. For example, in a prospective study of children presenting with severe malaria to Kilifi County Hospital in Kenya, on post-study genotyping we found that only 11 (0·5%) of 2220 children with severe malaria had SCA and that none had features of cerebral malaria.^17^ SCA might therefore protect against true cerebral malaria by protecting against high-density parasitaemia. Alternatively, protection might also relate to the reduced ability of *P falciparum*-infected SCA red blood cells to sequester in the microvasculature. A previous study found that the red cell surface molecule *P falciparum* erythrocyte membrane protein-1 (PfEMP1) was under-expressed on malaria-infected HbSS red cells and that this led to reduced cytoadherence.^37^ Subsequent work has linked this phenomenon to reduced protein trafficking through a disruption of the intracytoplasmic actin network.^38–40^ Finally, we recently showed that all children with SCA who do present with signs of severe malaria are infected by parasites of a specific genetic background.^41^ Another plausible explanation, therefore, is that such parasites are naturally less virulent. Further work will be required to pinpoint which of these mechanisms is most important in vivo.

To the best of our knowledge, ours is the second study to have assessed the burden of *P falciparum* parasites in children with SCA through the measurement of plasma PfHRP2 concentrations. One previous study involved children hospitalised with severe anaemia and malaria in Kampala, Uganda. The authors found that plasma PfHRP2 concentrations were significantly lower among 22 children who tested positive for SCA (197 ng/mL; IQR 6–469) than they were in 205 children with HbAA (1050 ng/mL; 468–2840; p<0·001).^42^ Although most of the children in our current study were recruited in areas where malaria transmission was considerably higher than in Kampala, overall concentrations of PfHRP2 were comparatively low, and were undetectable in almost a third of patients. We believe this finding was attributable to low-density infections rather than to *pfhrp2* deletions^43^ for several reasons. The primary method of malaria diagnosis in TRACT was by a PfHRP2-based RDT that would not have returned positive results when infections were caused by parasites with *pfhrp2* deletions. Furthermore, such RDTs primarily detect PfHRP2 in malaria-infected red blood cells, in which concentrations are approximately 20 times higher than they are in plasma, where they are mainly determined by the release of PfHRP2 at schizogony during the previous erythrocytic cycle.^14^ Finally, based on the current literature, the prevalence of parasites with *pfhrp2* deletions is still relatively low in most of sub-Saharan Africa, where they are generally found in children with asymptomatic infections,^43^ although either *pfhrp2* or *pfhrp2*/*3* deletions were found in almost 7% of parasites in one recent study, conducted in Uganda during the same era as TRACT.^44^ Moreover, the frequency was greatest in the east of the country, where the majority of TRACT participants were also recruited. Our observations should therefore be interpreted in this context. Our study materials did not include questions regarding specific treatments such as malaria chemoprophylaxis or the use of hydroxycarbamide among children recruited with known SCA, a potential limitation of our study. The possibility that some children might have been receiving specific treatments of this sort was our rationale for treating known and unknown SCA separately in our analysis. However, the fact that the clinical and parasitological indices of malaria were broadly similar in these two groups of children suggests that the receipt of specific therapies does not explain our current observations.

Although our study is consistent with the conclusion that children with SCA are innately protected from high- density malaria infections, this does not mean that in such children malaria cannot be a significant clinical problem. First, highlighting the major vulnerability of children with SCA to severe anaemia, almost 20% of all the children recruited to TRACT in Uganda who had no previous knowledge of their SCA status were subsequently found to have SCA. This is despite the fact that SCA affects less than 1% of children in the same population overall.^25^ Second, approximately one-third of the children with SCA tested positive for malaria overall, a likely trigger for their anaemic crises. This notion is supported by the fact that, unlike children with HbAA, haemoglobin concentrations were significantly lower in the malaria-positive subgroup of children with SCA compared with the malaria-negative subgroup. Finally, all the deaths that did occur in the malaria-positive subgroup were among children with detectable plasma concentrations of PfHRP2, presumably the group with the highest parasite burdens. Taken together, these observations reinforce how important it is that in Africa, SCA is diagnosed as early in life as possible and that affected children are offered malaria protection.

Some limitations to our analysis of the association of SCA and 28-day mortality are acknowledged. First, there is always some uncertainty in the causal interpretation of hazards ratios.^45^ Second, although we have attempted to adjust for all confounding factors, we cannot exclude the possibility that unmeasured confounders remain. Finally, in some groups, the number of events per variable in the multivariable model was lower than that which is generally recommended (ten),^46^ which in some situations could lead to biased estimates or poor confidence interval coverage.^47^

In conclusion, data from the current and other recent studies^17,25^ are consistent with an earlier model^6^ in which we proposed that even under conditions of intense malaria transmission, SCA affords a degree of innate resistance to high-density malaria infections, and thus protects against organ failure caused by microcirculatory obstruction by sequestered parasites. However, our study also shows that even low-density *P falciparum* infections can trigger sudden and catastrophic falls in haemoglobin that can rapidly result in death where emergency supplies of blood for transfusion are not available.^48^

## Supplementary Material

Supplementary Material

## Figures and Tables

**Table 1:** Baseline features of the malaria-positive Ugandan TRACT participants, stratified by sickle genotype

	HbAA (n=1815)	HbAS (n=63)	p value (HbAS *vs* HbAA)	Known SCA (n=167)	p value (known SCA *vs* HbAA)	Unknown SCA (n=180)	p value (unknown SCA *vs* HbAA)
Age, months	34 (18–56)	35 (20–50)	0·59	71 (45–98)	<0·0001	39 (19–63)	0·077
Sex							
Male	1017 (56%)	38 (60%)	0·52*	79 (47%)	0·034*	89 (49%)	0·10*
Female	798 (44%)	25 (40%)	..	88 (53%)	..	91 (51%)	..
Haemoglobin, g/dL	4·5 (3·6–5·4)	4·7 (3·6–5·6)	0·55	4·3 (3·4–5·1)	0·010	4·2 (3·5–4·9)	0·0004
MCV, fL	78 (73–84)	76 (71–83)	0·090	93 (85–104)	<0·0001	85 (78–95)	<0·0001
White blood cell count, 10^9^ cells per L	12 (8–19)	15 (9–22)	0·13	26 (18–36)	<0·0001	24 (16–34)	<0·0001
Platelet count, 10^9^ platelets per L	144 (82–245)	184 (114–272)	0·051	196 (116–315)	<0·0001	184 (115–266)	<0·001
CRP, mg/L	73 (38–128)	59 (25–86)	0·0059	42 (22–70)	<0·0001	32 (16–66)	<0·0001
Glucose, mmol/L	5·6 (4·8–6·3)	5·3 (4·7–6·4)	0·50	5·7 (5·2–6·4)	0·080	5·6 (4·9–6·3)	0·90
Lactate, mmol/L	2·8 (1·9–4·4)	2·7 (1·9–4·4)	0·59	2·5 (1·6–3·8)	0·0003	2·5 (1·8–3·7)	0·0024

Data are median (IQR) or n (%) unless stated otherwise. All data relate to the subgroup of children recruited to TRACT in Uganda who were malaria positive. p values were calculated using Fisher’s exact tests for categorical variables and Mann-Whitney U tests for continuous variables. SCA=sickle cell anaemia. MCV=mean corpuscular volume. CRP=C-reactive protein. *Global p values for sex overall.

**Table 2:** Markers of malaria parasite burden and severity by sickle genotype

	Uncomplicated stratum			Complicated stratum				
	HbAA (n=781)	Unknown SCA (n=84)	p value	HbAA (n=1034)	Known SCA (n=165)	p value (*vs* HbAA)	Unknown SCA (n=96)	p value (*vs* HbAA)
**Measures of parasite burden**								
*P falciparum* parasite density, parasites per μL*	39 400 (3534–138 580)	22 030 (7500–134 990)	0·89	32 120 (1485–147 400)	7860 (1923–40 860)	0·042	4022 (1162–32 820)	0·014
*P falciparum* parasites not detected	411 (53%)	64 (76%)	<0·0001	489 (47%)	106 (64%)	<0·0001	64 (67%)	0·0004
Plasma PfHRP2, ng/mL	448 (47–2125)	5 (0–29)	<0·0001	251 (11–2106)	8 (0–56)	<0·0001	8 (0–102)	<0·0001
PfHRP2 not detected	91 (12%)	35 (43%)	<0·0001	176 (17%)	66 (40%)	<0·0001	34 (39%)	<0·0001
**Features of severe malaria**								
Haemoglobin <4·0 g/dL	0	0	1·00	606 (59%)	69 (42%)	<0·0001	72 (75%)	0·0015
Hyperparasitaemia	110 (14%)	5 (6%)	0·041	154 (15%)	6 (4%)	<0·0001	3 (3%)	0·0005
PfHRP2 ≥1000 ng/mL†	296 (39%)	4 (5%)	<0·0001	345 (34%)	5 (3%)	<0·0001	4 (5%)	<0·0001
Cerebral malaria	5 (1%)	0	1·00	261 (25%)	4 (2%)	<0·0001	10 (10%)	0·0007
Convulsions	35 (4%)	0	0·041	126 (12%)	1 (1%)	<0·0001	7 (7%)	0·19
Severe malaria anaemia	80 (10%)	10 (12%)	0·58	218 (21%)	16 (10%)	0·0004	11 (11%)	0·024
Respiratory distress	0	0	1·00	262 (25%)	6 (4%)	<0·0001	9 (9%)	0·0002
Haemoglobinuria	0	0	1·00	330 (32%)	15 (9%)	<0·0001	17 (18%)	0·0036
Hypoglycaemia	3 (0·4%)	0	1·00	40 (4%)	3 (2%)	0·26	1 (1%)	0·25
Lactic acidosis	47 (6%)	3 (4%)	0·47	304 (29%)	21 (13%)	<0·0001	16 (17%)	0·0088
Renal impairment	3 (0·4%)	0	1·00	10 (1%)	0	0·37	1 (1%)	1·00

Data are median (IQR) or n (%) unless stated otherwise. p values were calculated using Fisher’s exact tests for categorical variables and Mann-Whitney U tests for continuous variables. The following definitions were used: hyperparasitaemia, >100 000 *P falciparum* parasites per μL; cerebral malaria, prostration in the presence of *P falciparum* parasitaemia of any density; severe malaria anaemia, a haemoglobin concentration of <5·0 g/dL in the presence of a *P falciparum* parasitaemia of >10 000 parasites per μL; respiratory distress, deep or rapid breathing in the presence of *P falciparum* parasitaemia of any density; hypoglycaemia, a blood glucose of <2·2 mmol/L; lactic acidosis, a whole blood lactate concentration of >5 mmol/L; renal impairment, blood urea nitrate >20 mmol/L. SCA=sickle cell anaemia. PfHRP2=*P falciparum* histidine-rich protein 2. *Parasite densities were only recorded for children with positive malaria slides: numbers with data in the uncomplicated stratum were HbAA 370/781 (47%) and unknown SCA 20/84 (24%), and numbers in the complicated stratum were HbAA 545/1034 (53%), known SCA 59/165 (36%), and unknown SCA 32/96 (33%). †This value has been used to discriminate likely true severe malaria from other severe illnesses in the presence of parasitaemia.^15,31^

**Table 3:** Mortality to day 28 among malaria-positive children by sickle genotype

	Deaths by 28 days	Kaplan–Meier mortality estimate (95% CI)	Hazard ratio (95% CI)	p value
HbAA	52/1815	2% (2–3)	1 (reference)	··
HbAS	2/63	3% (1–12)	2·72 (0·63–11·75)	0·18
Known SCA	3/167	2% (1–5)	1·07 (0·31–3·76)	0·11
Unknown SCA	2/180	1% (0–4)	0·67 (0·15–2·90)	0·59

Results from Cox proportional hazards models from date of randomisation to date of death, censored at day 28, or date last known to be alive if vital status at end date was unknown. The model included adjustments for transfusion volume (30 mL/kg or 20 mL/kg), temperature, and the interaction between the two, modelled with natural cubic splines (five knots at 36·0, 36·8, 37·3, 37·9, and 39·2), site, HIV positivity, oxygen saturation, blood group, respiratory rate, Blantyre Coma Score, fitting at admission, and lactate. The development and use of the model was as previously described.^20^ A global test of the proportional hazards assumption had p=0·33, with p>0·05 for all variables with the exception of one temperature spline which had p=0·036. 11 participants were censored (ten lost to follow-up, one withdrew). SCA=sickle cell anaemia.

## Data Availability

The TRACT trial data are held at the MRC Clinical Trials Unit at University College London (London, UK), which encourages optimal use of data by employing a controlled access approach to data sharing, incorporating a transparent and robust system to review requests, and providing secure data access consistent with the relevant ethics committee approvals. All requests for data are considered and can be initiated by contacting mrcctu.ctuenquiries@ucl.ac.uk.

## Research in context

### Evidence before this study

We searched PubMed for articles on the relationship between sickle cell anaemia (SCA) and malaria using the search terms (“sickle cell disease” OR “sickle cell anaemia” OR “sickle cell anemia”) AND “malaria”. The search was done on June 16, 2022, without date or language constraints. Our search revealed 865 articles in total, of which 671 included original research. Most studies included very small numbers of children with both SCA and malaria and less than ten were relevant to the question at hand. It is widely believed that Plasmodium falciparum malaria has exerted opposite selective forces on children with sickle cell trait (HbAS) and SCA (HbSS). Although the evidence for a malaria-protective effect of HbAS is now overwhelming, recent epidemiological studies have raised questions about the negative effects of malaria on children with SCA.

### Added value of this study

To our knowledge, the current study, which included 347 children presenting with both SCA and malaria to three hospitals in Uganda, is the most comprehensive study yet conducted on this subject. Unlike most previous studies, the current study includes the measurement of plasma concentrations of *P falciparum* histidine-rich protein 2 (PfHRP2), an assay that captures the total body load of *P falciparum* parasites in patients with malaria. Recent studies have shown that such measurements can be useful in distinguishing children hospitalised with true severe malaria from children with severe febrile illnesses of alternative cause that are accompanied by incidental parasitaemia.

### Implications of all the available evidence

In conjunction with the findings from previous epidemiological studies, the current study provides strong evidence in support of a malaria-protective effect of SCA at a level similar to that afforded by HbAS. Nevertheless, the study also shows that this protection is incomplete, and that even low-grade malaria infections can precipitate severe anaemic crises in children with SCA that, in the absence of blood transfusion, would almost certainly be rapidly fatal. Overall, the study supports the need for early diagnosis and proactive interventions for preventing malaria in children with SCA.
