# Supplementary material for: Sickle cell anaemia and severe Plasmodium falciparum malaria: a secondary analysis of the Transfusion and Treatment of African Children Trial (TRACT)
Source: Lancet Child Adolesc Health. 2022 Sep;6(9):606–13. doi: 10.1016/S2352-4642(22)00153-5 (PMC7613576; doi:10.1016/S2352-4642(22)00153-5)
Supplement: Supplementary appendix [file mmc1.pdf]

THE LANCET
Child & Adolescent Health

Supplementary appendix

This appendix formed part of the original submission and has been peer reviewed. We post it as supplied by the authors.

Supplement to: Uyoga S, Olupot-Olupot P, Connon R et al. Sickle cell anaemia and severe *Plasmodium falciparum* malaria: a secondary analysis of the Transfusion and Treatment of African Children Trial (TRACT). *Lancet Child Adolesc Health* 2022; published online July 1. https://doi.org/10.1016/S2352-4642(22)00153-5.

Supplementary Table 1. Malaria positive children with undetectable plasma PfHRP2 by method of malaria diagnosis

	AA	AS	Known SS	Unknown SS	Total
Positive by both RDT and microscopy	50/873 (6%)	6/30 (20%)	17/55 (31%)	12/46 (26%)	85/1004 (8%)
Positive by RDT, negative by microscopy	211/879 (24%)	11/32 (34%)	48/105 (46%)	56/120 (47%)	326/1136 (29%)
Other*	6/18 (33%)	-	2/6 (33%)	1/2 (50%)	9/26 (35%)

*Includes: 10 children negative on RDT but positive by slide; 4 children positive by RDT, slide not done; 12 children positive by slide but RDT not done/invalid.

Supplementary Table 2. Outcomes by SCA genotype and plasma PfHRP2 level.

	PfHRP2 (ng/mL)	AA	AS	P-AS vs AA	Known SCD	P-Known vs AA	Unknown SCD	P-Unknown vs AA
**Mortality - 7 days**	<10	7/361 (2%)	0/22 (0%)	0.66	0/85 (0%)	0.36	0/91 (0%)	0.35
	10-100	7/282 (2%)	1/13 (8%)	0.31	3/48 (6%)	0.17	0/42 (0%)	0.60
	100-1000	6/486 (1%)	0/13 (0%)	1.00	0/28 (0%)	1.00	0/27 (0%)	1.00
	>1000	18/641 (3%)	1/14 (7%)	0.34	0/5 (0%)	1.00	2/8 (25%)	0.02
**Mortality - 28 days**	<10	12/361 (3%)	0/22 (0%)	1.00	0/85 (0%)	0.13	0/91 (0%)	0.14
	10-100	8/282 (3%)	1/13 (8%)	0.34	3/48 (6%)	0.20	0/42 (0%)	0.60
	100-1000	9/486 (2%)	0/13 (0%)	0.79	0/28 (0%)	1.00	0/27 (0%)	1.00
	>1000	21/641 (3%)	1/14 (7%)	0.38	0/5 (0%)	1.00	2/8 (25%)	0.03

Supplementary Table 3. The distribution of PfHRP2 values by SCA group.

**PfHRP2 (ng/mL)**	**AA (n=1,770)**	**AS (n=62)**	**Known SCA (n=166)**	**Unknown SCA (n=168)**
0	267 (15%)	17 (27%)	67 (40%)	69 (41%)
>0 - <50	289 (16%)	16 (26%)	52 (31%)	57 (34%)
50 - <100	87 (5%)	2 (3%)	14 (8%)	7 (4%)
100 - <500	339 (19%)	7 (11%)	26 (16%)	23 (14%)
500 - <1000	147 (8%)	6 (10%)	2 (1%)	4 (2%)
1000 - <2000	187 (11%)	5 (8%)	2 (1%)	2 (1%)
2000+	454 (26%)	9 (15%)	3 (2%)	6 (4%)

Supplementary Figure 1. Study flow for the TRACT trial overall.

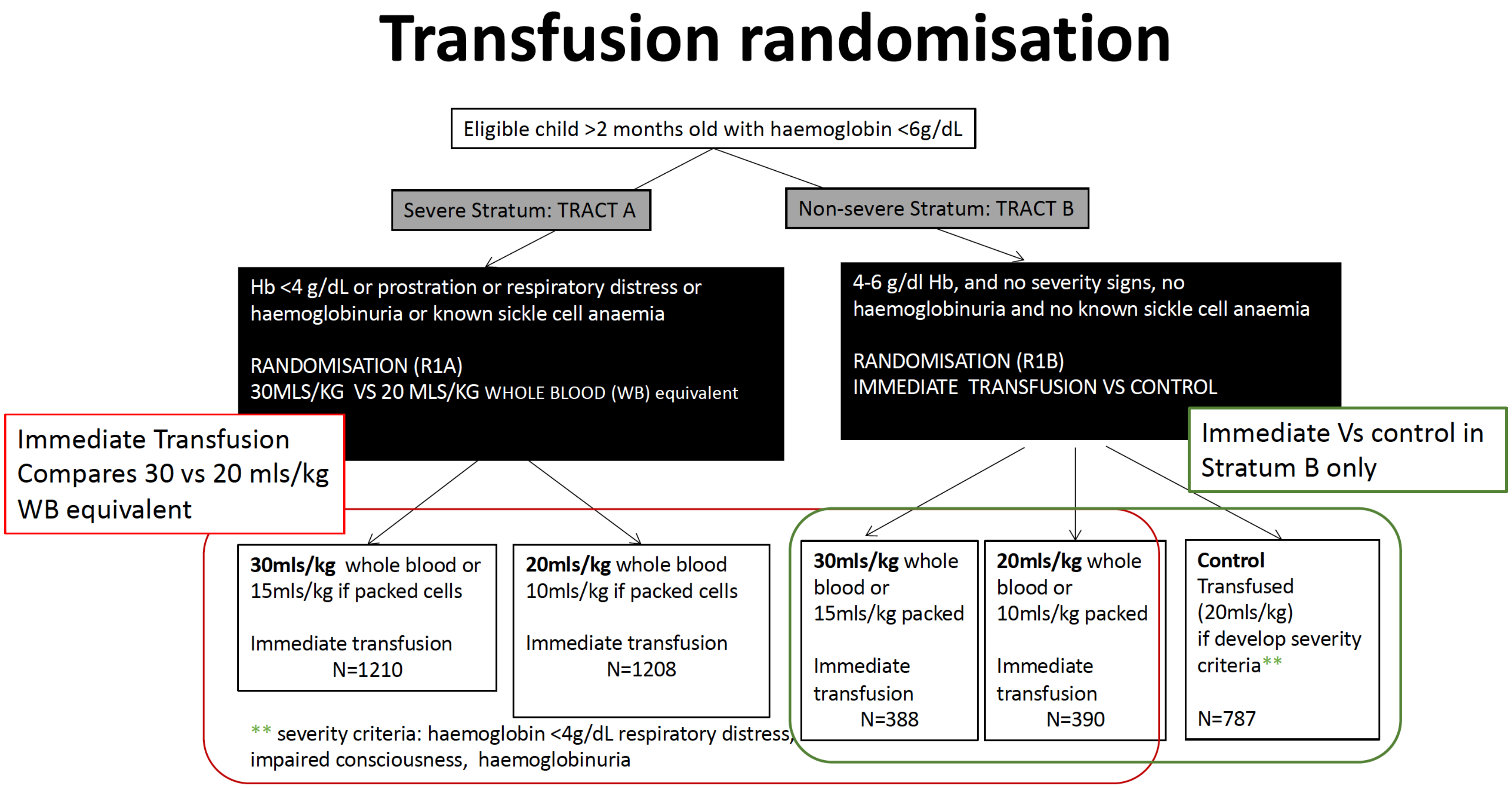

Supplementary Figure 2. Plasma PfHRP2 in children by SCA genotype.

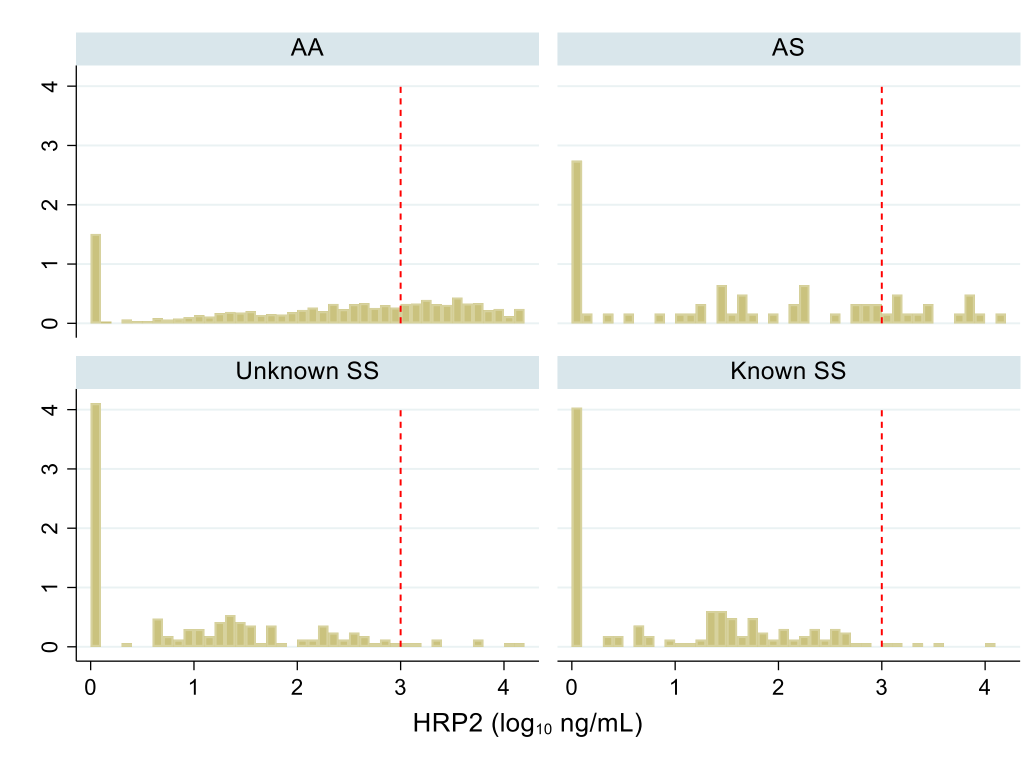

Dashed red lines denote the threshold value for severe malaria.
